# Polymorphic eruption of pregnancy associated with bullae formation

**DOI:** 10.1016/j.jdcr.2023.10.003

**Published:** 2023-10-15

**Authors:** Frank F. Zhou, Kyle Cheng, Emily C. Boozalis, Gregory A. Gates

**Affiliations:** a David Geffen School of Medicine at University of California, Los Angeles, California; b Division of Dermatology, Department of Medicine, University of California, Los Angeles, California; c Department of Pathology and Laboratory Medicine, University of California, Los Angeles, California

**Keywords:** bullae, polymorphic eruption of pregnancy, pruritic urticarial papules and plaques of pregnancy

## Introduction

Polymorphic eruption of pregnancy (PEP) is a common and benign inflammatory skin disorder that usually affects primigravidas in the third trimester of pregnancy. It typically presents as pruritic, erythematous papules within abdominal striae, which then coalesce into plaques in the trunk and extremities.^1^ In about half of patients, polymorphic features such as eczematous lesions, tiny vesicles, or targetoid lesions can develop in the later stages of disease.^2^ However, to our knowledge, the presence of bullae in PEP has only been described once previously.^3^^,^^4^ Here, we present a highly unusual case of PEP associated with late bullae formation.

## Case report

**Fig 1:**
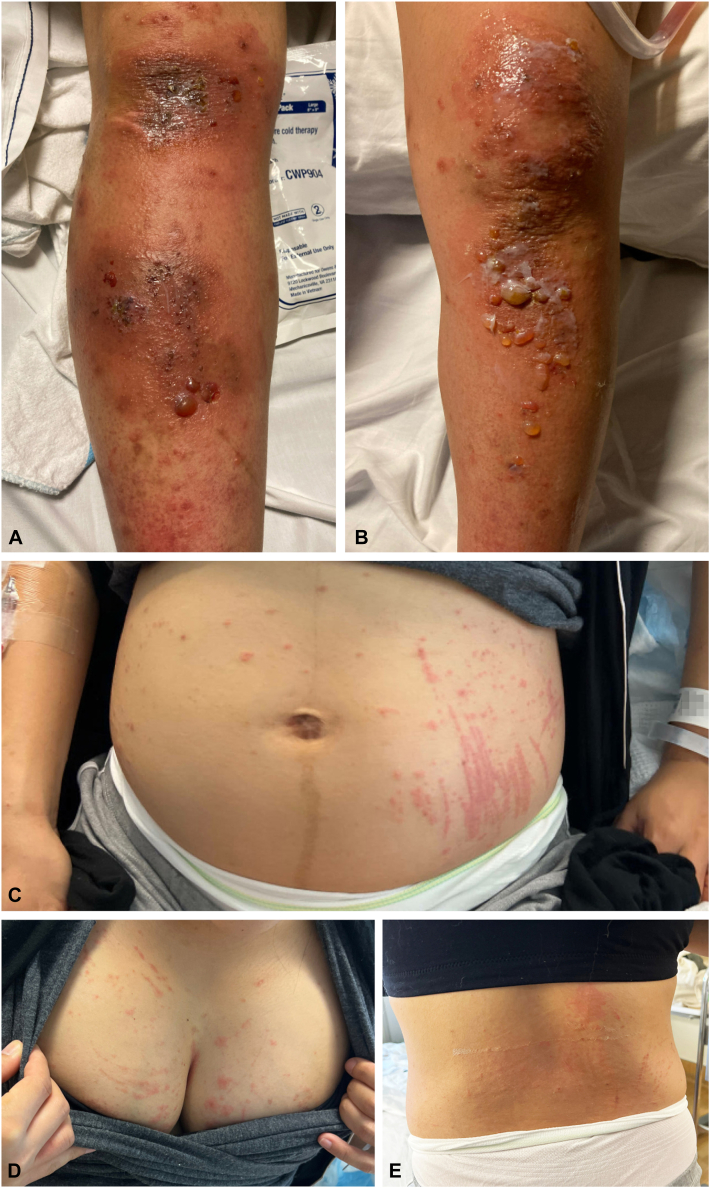
**A,** Right lower extremity and (**B**) left lower extremity demonstrating erythematous, dusky plaques with overlying tense bullae on the legs and knees. **C,** Abdomen, (**D**) chest, and (**E**) back demonstrating pruritic, erythematous papules.

A 37-year-old primigravid female who was postpartum day 0 status after full-term spontaneous vaginal delivery presented with a 2-week history of highly pruritic, erythematous papules across the abdomen, back, chest, and lower extremities sparing the umbilicus. There were also erythematous, dusky plaques and overlying tense bullae on the bilateral lower legs and knees that had developed from the papules 1 week ago (Fig 1). She had been prescribed oral cephalexin and topical mupirocin for potential cellulitis by urgent care and topical triamcinolone without improvement. No warmth, pain, or purulent discharge was noted at any site, and her extremities had no edema. The patient denied any history of fever or chills. She denied any recent outdoor exposure to plant or irritant and had no new topical product or cosmetic usage. She also had no past medical or dermatologic history, including no history of contact dermatitis, and no known allergies.

**Fig 2:**
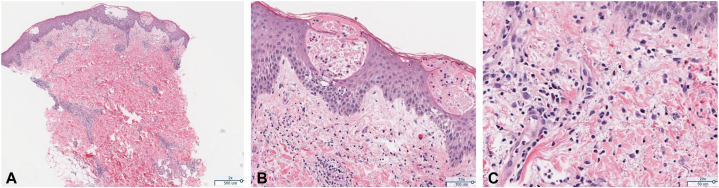
Punch biopsy of the right lower extremity (**A, B,** and **C,** Hematoxylin-eosin stain; original magnifications: **A,** ×2; **B,** ×10; and **C,** ×20) showing intraepidermal and subcorneal vesicle formation with associated eosinophils and lymphocytes and superficial papillary dermal edema.

Punch biopsy of the right lower extremity demonstrated intraepidermal and subcorneal vesicle formation with associated eosinophils and lymphocytes, superficial papillary dermal edema and red cell extravasation, and perivascular interstitial lymphohistiocytic inflammation (Fig 2). The Gram/periodic acid–Schiff with diastase special stain was negative for pathogenic organisms. Direct immunofluorescence (DIF) testing for IgG, IgG4, IgM, IgA, and C3 and basement membrane BP180 NC16A antibody indirect immunofluorescence tests were negative.

The clinical history, physical examination, and laboratory findings established a diagnosis of PEP. The negative DIF and NC16A antibody results excluded a diagnosis of pemphigoid gestationis. The lack of warmth, pain, purulent discharge, fever, chills, response to recent antibiotics, and bacteria on Gram stain excluded a diagnosis of superimposed cellulitis or erysipelas. The lack of recent exposure to any common irritant and the unusual and widespread distribution of lesions did not favor a diagnosis of superimposed contact dermatitis.

The patient was treated with 1 month-long prednisone taper, starting at 40 mg daily, and clobetasol 0.05% ointment twice daily. Antihistamines were given for symptomatic relief. The patient reported significant improvement in her lesions after 10 days.

## Discussion

This case highlights that bilateral lower extremity bullae formation can be a possible late polymorphic feature of PEP, also known as pruritic urticarial papules and plaques of pregnancy. Polymorphic features are seen in about half of patients with later-stage PEP, but they are typically limited only to eczematous lesions, tiny vesicles, or targetoid lesions.^2^ To our knowledge, PEP with bullae formation has only been reported once previously.^3^^,^^4^ In that report, the patient first developed small vesicles on both forearms, which later converted to bullae 2 days after cesarean section.^3^

PEP is a common condition, affecting as many as 1 in 130 pregnancies.^5^ The etiology remains unclear, but excessive abdominal skin stretching has been hypothesized to be contributory, especially because PEP occurs more commonly with twin or triplet gestations.^4^

The diagnosis of PEP can be made clinically in a third trimester patient with pruritic plaques on the abdomen that coalesce into papules, often also affecting the extremities, chest, and/or back.^6^ In PEP, the umbilicus, face, palms, soles, and mucosa are nearly always spared. Histology typically reveals a perivascular and/or interstitial lymphocytic infiltrate alongside eosinophils.^7^

The presence of bullae in our patient raised the possibility of other etiologies, most notably pemphigoid gestationis. However, both DIF testing of the basement membrane and BP180 NC16A indirect immunofluorescence testing are highly sensitive and specific for pemphigoid gestationis, and neither was positive in our patient.^8^ This case thus highlights that bullae formation in a pregnant patient does not necessarily indicate an autoimmune process such as pemphigoid gestationis but may be secondary to PEP instead.

Bullae can also be seen in other autoimmune diseases, severe cellulitis, contact dermatitis, and drug eruption, among other etiologies. However, these were found to be less consistent with our patient’s presentation than a diagnosis of PEP.

Specifically, bullous pemphigoid and pemphigus vulgaris were excluded given our patient’s young age, incompatible histopathology findings, and negative DIF testing. Severe cellulitis was excluded given the lack of warmth, pain, purulence, fever, and response to antibiotics. Contact dermatitis could not be entirely excluded histologically but was deemed unlikely given no history of recent exposure to irritants, no history of prior contact dermatitis, and an unusual distribution of lesions. Drug reaction was excluded because our patient had not started any new medication before bullae formation. Edema and stasis bullae were excluded given the patient’s lack of peripheral edema and inconsistent pathology. Stasis dermatitis with id reaction was excluded because no stasis changes, such as vascular proliferation or hemosiderin, were seen on histopathology, and id reactions typically do not produce bullae. Finally, although the linear lesions of the abdomen may prompt consideration of dermatographism, this was excluded because our patient had a 2-week history of plaques, the lesions were not shifting or changing, and there was negative dermatographism on physical examination. In fact, the Koebner response and correspondingly linear lesions in the abdominal striae are a common occurrence with PEP and thus consistent with our diagnosis.^9^^,^^10^

Treatment of PEP can involve topical moisturizers for symptom relief in mild cases, sedating antihistamines as adjunct therapy for pruritus and sleep, and topical or systemic corticosteroids for severe cases.^4^ The vast majority of PEP cases resolve within days to weeks. There is no increased risk of morbidity to the mother or child associated with the disease or treatment, and recurrence is rare.^4^

## Conflicts of interest

None disclosed.
